# Dicistronic MLV-retroviral vectors transduce neural precursors *in vivo *and co-express two genes in their differentiated neuronal progeny

**DOI:** 10.1186/1742-4690-2-60

**Published:** 2005-09-29

**Authors:** Edmund A Derrington, Marcelo López-Lastra, Jean-Luc Darlix

**Affiliations:** 1 LaboRétro, INSERM U412, Ecole Normale Supérieure de Lyon, 46 Allée d'Italie, Lyon 69364 Cedex 07, France; 2 Laboratorio de Virología Molecular, Centro de Investigaciones Médicas, Pontificia Universidad Católica de Chile, Marcoleta 391, Santiago, Chile

## Abstract

Dicistronic MLV-based retroviral vectors, in which two IRESes independently initiate the translation of two proteins from a single RNA, have been shown to direct co-expression of proteins in several cell culture systems. Here we report that these dicistronic retroviral vectors can drive co-expression of two gene products in brain cells *in vivo*. Injection of retroviral vector producer cells leads to the transduction of proliferating precursors in the external granular layer of the cerebellum and throughout the ventricular regions. Differentiated neurons co-expressing both transgenes were observed in the cerebellum and in lower numbers in distant brain regions such as the cortex. Thus, we describe an eukaryotic dicistronic vector system that is capable of transducing mouse neural precursors *in vivo *and maintaining the expression of genes after cell differentiation.

## Background

The brain constitutes one of the most important organs for gene therapy. Considerable interest resides in the development of vector-based therapies for many of the brain diseases, either to allow the expression of exogenous genes to compensate for a metabolic deficit, to express a growth factor and thus inhibit neural degeneration or to target suicide genes to cancer cells. An alternative approach has been the development of cellular vectors [1-3]. Uncommitted neural precursor cells can be isolated, transduced and grafted into host brains. They adapt to novel environments by stable integration and the expression of location-appropriate phenotypes in host. This opens new avenues for the use of neural stem cells as cellular vectors for gene therapy in the central nervous system (CNS) [2-6]. Both endogenous and transplanted stem cells spontaneously migrate to the site of lesions where they integrate to repopulate the damaged tissue [7-9].

Retroviral vectors based on the γ-retrovirus murine leukemia virus (MLV) are of particular interest for the transduction of neural precursor cells either *ex vivo *to generate cellular vectors, or *in vivo *to directly target endogenous neural precursors. They specifically target proliferating cells [10], integrate into the host genome and are conserved in cellular progeny [11]. This has made MLV-vectors a tool of choice to trace lineages and assess the function of specific genes in rodent CNS *in vivo *[12-14]. In previous studies we established that dicistronic MLV-based retroviral vectors efficiently transduced cells derived from a transformed human neural stem cell line [15], or cells from a primary culture of neural precursors [16]. Here we report that dicistronic MLV-based vectors can deliver and maintain expression of marker genes during neural differentiation in the CNS of new born mice.

Vector producer cells were injected in the region of the developing cerebellum, where the generation of neurons from proliferating precursors continues after birth [17-21]. At early periods post-injection, transduced cells were observed in the external granular layer (EGL) of precursors and migrating towards the internal granular layer (IGL). At later time differentiated neurons were observed scattered about the IGL or in patches. Analysis of other brain regions demonstrated a large number of transduced cells in the ependymal walls throughout the ventricular system and in the subventricular zone. Thus, our results show that dicistronic MLV-based vectors co-expressing two marker transgenes, human placental alkaline phosphatase (PLAP) and neomycin phosphotransferase (Neo) [22,23], transduce proliferating neural precursors *in vivo *and can penetrate throughout the ventricular system when producer cells are grafted to host animals. Moreover, transduced neural precursors maintain expression of both transgenes after differentiation into neurons demonstrating that the activity of both internal ribosome entry segments (IRES) used in their design is not altered *in vivo *by neural differentiation.

## Results

### Location of MLV-vector producer cells

**Figure 1:**
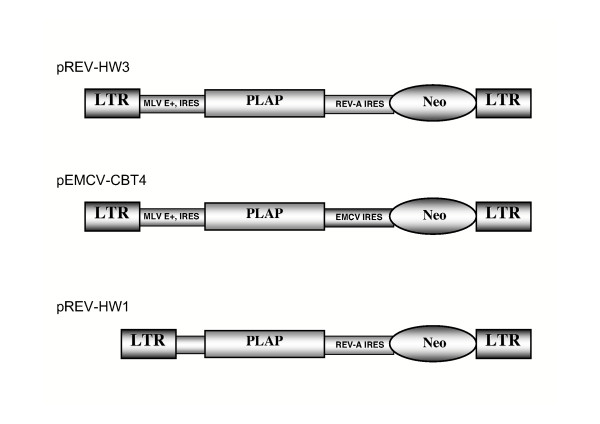
**Schematic representation of dicistronic MLV vectors**. Vectors used in this study have been previously described [22, 23]. MLV E+ corresponds to the enhanced packaging region of MLV and the internal ribosome entry signal (IRES). pREV-HW3 and pEMCV-CBT4 contain two IRESes [22, 23]. For both vectors the first is that of MLV and drives expression of human placental alkaline phosphatase (PLAP). In the pREV-HW3 the second IRES is that of REV-A [22], while in pEMCV-CBT4 it is that of EMCV [66]. In both vectors the second IRES drives the expression of neomycin phosphotransferase (Neo). The pREV-HW1 lacks the packaging sequence and the IRES of MLV and thus it cannot generate recombinant virus [22]. The pREV-HW1 missing the MLV Psi/IRES sequences was used as an internal negative control.

**Figure 2:**
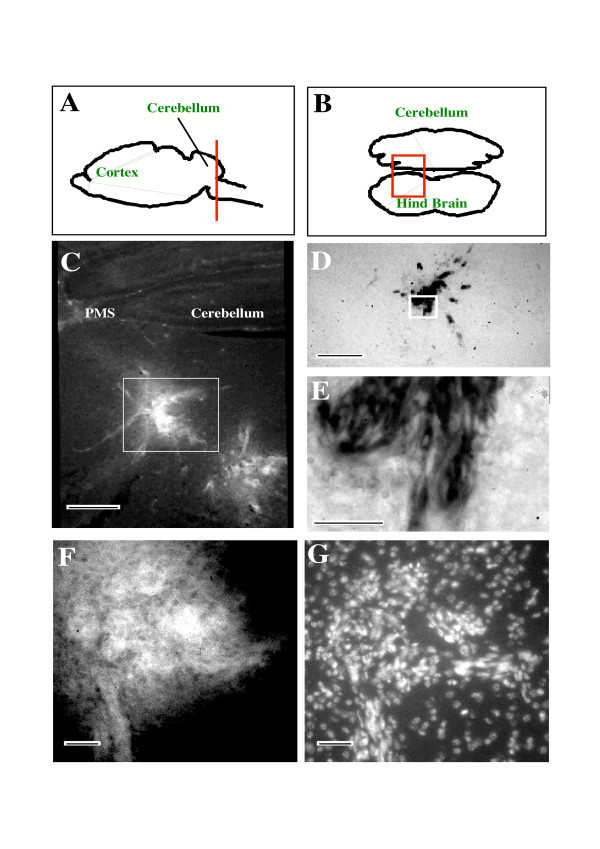
**Location of injected MLV-vector producer cells**. Injected helper cells were found mainly in the hind brain beneath the caudal cerebellum. The red line in A indicates the position in the rostro-caudal axis, and the red rectangle in B shows the location of the photomicro graph presented in C. The sections in D, F and G correspond to the region bordered by the white rectangle shown in C. E shows the region bordered by the white rectangle in D at higher magnificationInjected cells were easily distinguished from the surrounding tissue on the basis of transgene expression shown by immunofluorescence for Neo (C) or PLAP (F). Brightly stained cells express transgene. Alternatively PLAP activity could be revealed by histochemistry in which case the dark cells express PLAP (D and E). It was also possible to identify injected producer cells by staining DNA as shown in field G which corresponds to the same field as D stained with bis-benzimide. The nuclei of injected cells appeared to fluoresce more brightly than brain cell nuclei and were elongated in contrast to the round nuclei typical of neural cells. Macroscopically, injected cells appeared as disorganized patches in the surrounding brain parenchyma. PMS, perimedian sulcus. Scale bars indicate 200 μm (C and D), 20 μm (E, F and G)

**Figure 3:**
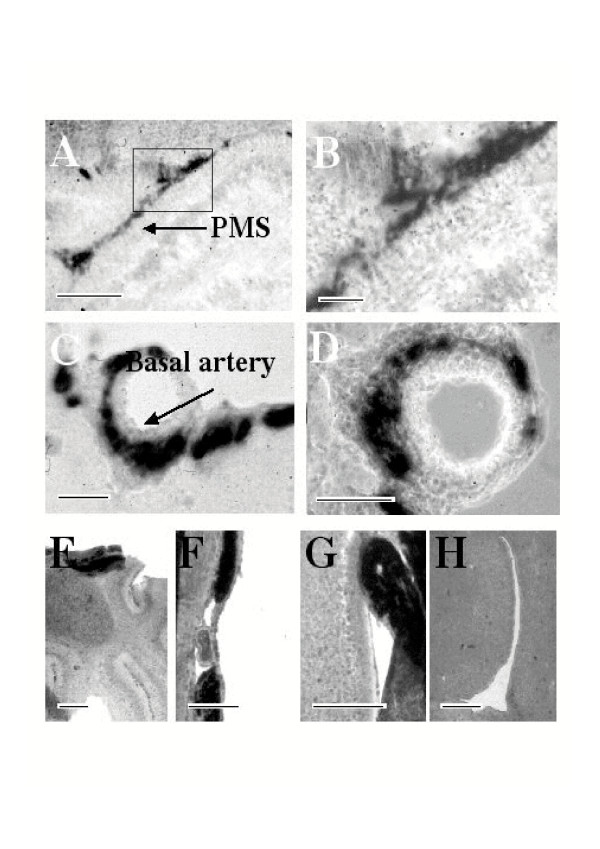
**Dissemination of injected MLV vector producer cells from the injection site**. Panels A, B, C and D are micrographs from brain sections of an animal injected with pREV-HW3 vector producer cells, while panels E, F, G and H are micrographs from brain sections of an animal injected with helper cells transfected with pREV-HW1. Panel B shows the region of A bordered by a black rectangle at greater magnification. PLAP histochemical staining shows cells in different sites including the 4^th ^ventricle and its lateral recesses and in the perimedian sulcus (A, B and G, respectively) and trapped in the subarachnoid space, between the meninges on the surface of the brain parenchyma, particularly in the region of the basal artery (C, D and F). Similar distributions were obtained with vector producing cells (A, B, C and D) and helper cells transfected with pREV-HW1 (E, F, G and H). PMS, perimedian sulcus. Size bars indicate 200 μm (A and G), 100 μm (B), 125 μm (C and D), 450 μm (E), 360 μm (F) and 250 μm (H).

The cerebellum of newborn mice constitutes an accessible model system and was used as target to evaluate the capacity of dicistronic MLV retroviral vectors (Fig. 1) to transduce neural precursors *in vivo*. At early ages the cartilage of the skull has not undergone calcification, is very thin and can be easily pierced with a needle; thus surgery is not required prior to injection in the brain parenchyma. At this early stage however, stereotactic guidance of the injection needle is not practical because of the difficulty of maintaining a newborn mouse head in a steady coordinated position. Therefore, upon injection of recombinant virus producing cell lines it was important to determine the location of the cells at different times post-injection. The principal site where producer cells were found 5 days post injection (dpi) corresponded to the hindbrain beneath the cerebellum (Fig. 2) with smaller clusters of cells following the needle trace up to the surface of the brain. The micrographs shown (Fig. 2C to 2G) were produced from adjacent coronal sections of caudal cerebellum and the underlying hindbrain, in the regions indicated (Figure 2A and 2B). Injected cells were easily distinguished from the surrounding tissue on the basis of transgene expression shown by immunofluorescence for Neo (Fig. 2C) or PLAP (Fig. 2F). PLAP activity was also revealed by histochemistry (Fig. 2D and 2E). Injected producer cells were also identified by staining DNA (Fig. 2G) because their nuclei appeared to fluoresce more brightly than brain cell nuclei and were elongated as opposed to the round nuclei typical of neural cells. Injected producer cells were not restricted to the injection site since histochemically labelled producer and control cells were also found at different sites including the 4^th ^ventricle and its lateral recesses and in the perimedian sulcus (Fig. 3A,B and 3G; PLAP staining) and trapped in the subarachnoid space, between the meninges on the surface of the brain parenchyma, particularly in the region of the basal artery (Fig. 3C,D and 3F). Similar distributions were obtained with pREV-HW3 vector producing cell lines (Fig. 3A,B,C and 3D), and control helper cells transfected with pREV-HW1 (Fig. 3E,F,G and 3H). The latter vector lacks the viral packaging sequence and is thus not incorporated into recombinant vector particles [22]. The absence of stained cells in the region of the lateral ventricles indicates the failure of graft cells to migrate such great distances upstream of the injection site (Fig. 3H). Taken together these observations are consistent with a significant infiltration of injected cells into the cerebrospinal fluid (CSF) and their being carried via the CSF throughout the ventricular system and into the subarachnoid space. At later times (10 dpi and later) producer cells were no longer observed. We do not know if this is because of the suboptimal conditions for their survival in the brain or whether they were actively eliminated. The latter hypothesis appears less likely because at this early stage in postnatal life immunotolerance of self is still being acquired. Furthermore the brain is an immunoprivileged organ well isolated from the immune system. Lastly, we did not identify any evident signs of inflammation such as activated macrophages or infiltrating lymphocytes in the brain parenchyma.

### Transgene expression in differentiated cerebellar cells *in vivo*

**Figure 4:**
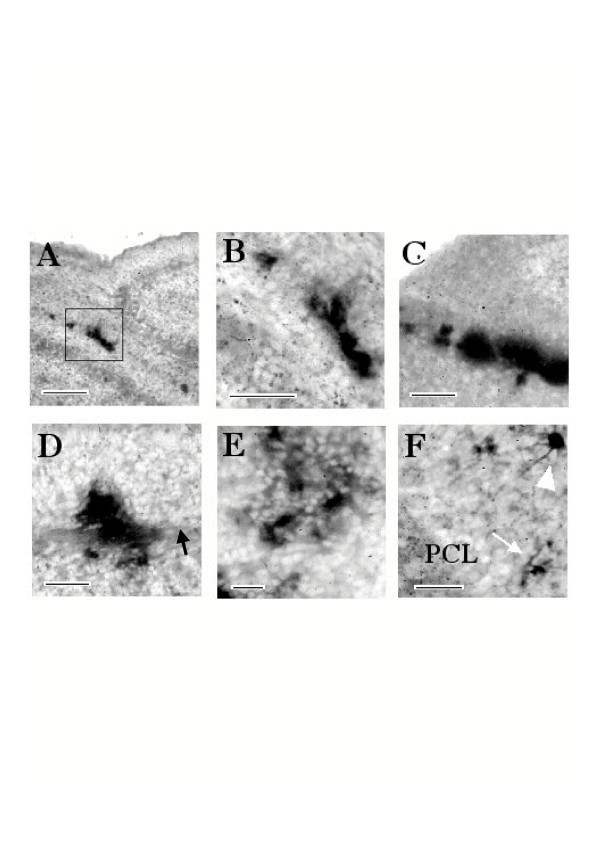
***In vivo *transduction of cells using pREV-HW3 vector in the cerebellum**. 4 days post injection, histochemical staining of PLAP in brain sections shows labeled cells in the region occupied by neural precursors in the external granular layer of the cerebellum, observed as discrete cells (A and B) or patches of staining (C). Panel B shows the region bordered by the black rectangle in A at higher magnification. 15 dpi histochemistry revealed transduced cells in patches and scattered about the parenchyma of the cerebellum (D, E and F). The majority of cells were found in the internal granular layer (E and F) but cells were also found in and astride bands of cerebellar white matter which is indicated by a black arrow (D). Discrete cells often exhibit a clearly neuronal morphology (F), the thin arrow indicates a cell with the morphology of a granular neuron, the arrow head indicates a cell with the morphological characteristics of a cerebellar golgi neuron. PCL, purkinje cell layer. Scale bars are 200 μm (A), 100 μm (B, C, D), and 40 μm (E, F)

We next sought to determine the location of cells transduced by the dicistronic MLV vector *in vivo*. At 4 dpi histochemical staining for the PLAP, reporter gene under the control of the MLV IRES, revealed transduced cells mainly in the EGL (Fig. 4A and 4B). Labeled cells in the EGL were often clustered along the edge of the parenchyma of the cerebellum and were morphologically fusiform (Fig. 4A and 4B), however patches of staining were also observed in which it was difficult to distinguish discrete cells (Fig. 4C). At 15 dpi PLAP histochemical staining revealed transduced cells in patches and scattered about the parenchyma of the cerebellum (Fig. 4D,E and 4F). The majority of cells were found in the IGL (Fig. 4E and 4F) but cells were also found in and astride bands of cerebellar white matter (Fig. 4D). The high density of the labeling made it difficult to attribute a phenotype to individual cells on the basis of morphological characteristics. However, on the basis of their locations in fibre tracts and in the IGL, PLAP expressing cells probably included both neurons and glia. In the regions of the IGL where labeled cells were more parsimoniously scattered it was possible to distinguish cells with a clearly neuronal morphology (Fig. 4F).

**Figure 5:**
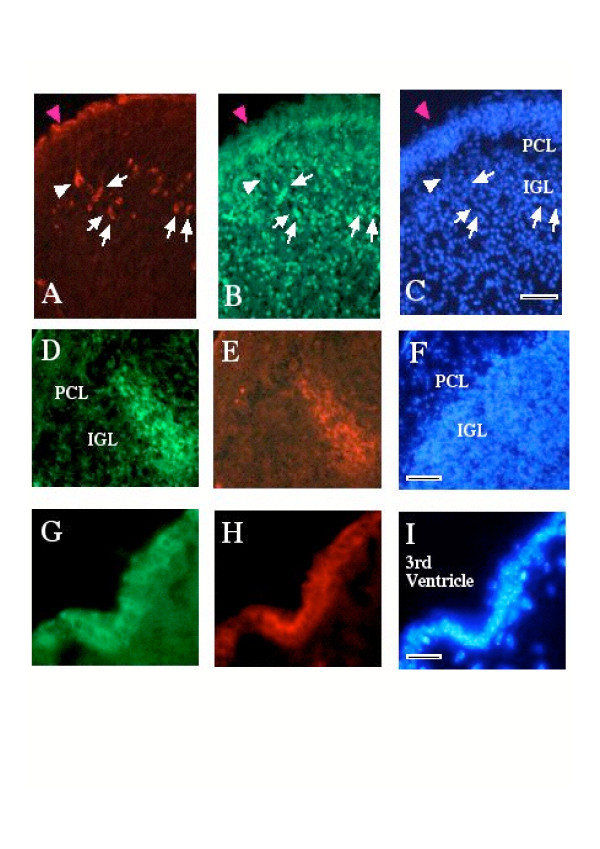
***In vivo *transduction of neural cells**. Immunohistochemical labeling for PLAP (A) and the neuron-specific HU antigen (B) was used to identify pREV-HW3 transduced neurons in the cerebellum (5 dpi). Examples of cells expressing both antigens are indicated by white arrows. Cells expressing PLAP were identified on the peripheral extremity of the section (magenta arrowhead in A, B and C). Transduced cells were also found in the brain parenchyma (example indicated by white arrowhead). In all the regions examined transduced cells co-expressed both proteins as illustrated by immunodetection of both PLAP (D) and Neo (E) in cells in the IGL of the cerebellum (14 dpi). Co-expression of both antigens, PLAP (G) and Neo (H), also occurred in cells in the ventricular region such as in the ependymal walls of the 3rd ventricle (G, H and I). DNA is stained with bis-benzimide (C, F and I). PCL, purkinje cell layer; IGL, internal granular layer. Scale bars correspond to 150 μm (C and F), 75 μm (I)

Immunohistochemistry for PLAP revealed intensely labeled cells in the IGL (Fig. 5A). An antiserum directed against the HU antigen which is specifically expressed only by post-mitotic neurons in the brain [24,25] allowed the unambiguous identification of some of the PLAP expressing cells as neurons (Fig. 5B, arrows). Cells expressing PLAP were also identified on the peripheral extremity of the section (Fig. 5A,B and 5C, magenta arrowhead), however, HU staining in this region is at background levels. These stained cells, at this stage (5 dpi), may correspond to undifferentiated precursors, producer cells or transduced meningeal cells. At 15 dpi double labeling for PLAP and Neo revealed patches of cells in the internal granular layer that expressed both transgenes (Fig. 5D,E and 5F). Similar data where obtained when vector pEMCV-CBT4 was used (data not shown) indicating that the MLV-based double IRES vectors can direct expression of two distinct gene products in cerebellar neurons.

Taken together these results show that MLV-IRES vectors are able to transduce precursor cells in the CNS *in vivo *and that the IRESes of different viruses such as MLV, EMCV and REV-A remain functional in differentiated neurons in the animal.

### Transduction of cells in different brain regions

**Figure 6:**
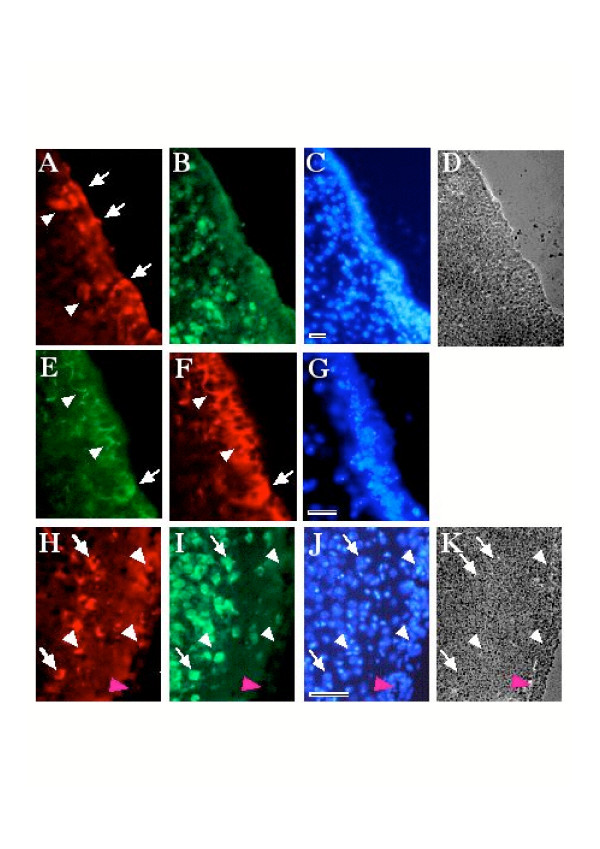
***In vivo *transduction of cells in other brain regions**. Immunohistochemical staining reveals pREV-HW3 transduced cells in and adjacent to the ependymal walls throughout the ventricular system including in the lateral ventricles (A, B, C and D). PLAP expressing cells are identified in the ependymal wall, and in the adjacent brain parenchyma (arrow heads in A). Transduced cells are not reactive to anti-HU antibody (B). Double labeling for PLAP (E) and GFAP (F) showed that the PLAP transgene predominantly co-localizes with GFAP in cells (arrow in E and F) and processes (arrow heads). Analysis of the overlying cortex showed cells co-expressing PLAP (H) and HU (I) in the most superficial layers of cortex (arrows in H and I). Many of the transduced cells in this region did not express HU (white arrow heads in H, I, J and K). Note the absence of HU staining in small piece of attached meninges (magenta arrow head in H, I, J and K). DNA staining with bis-benzimide is shown C, G and J and phase contrast of the section in A, B and C, and the section in H, I and J, are shown in D and K, respectively. Scale bars correspond to 75 μm (C, G) and 100 μm (J).

Although the primary target for transduction was the cerebellum the spread of the dicistronic MLV vector to neural cells in other brain regions was also evaluated. Numerous transduced ependymal cells were observed in the ventricular walls and in the 3^rd ^and 4^th ^ventricles (Fig. 5G,H and 5I). A significant sub-population of transduced cells was also observed in the lateral ventricles (Fig. 6A–G) a site very distant from the site of injection of the producer cells. Whereas many transduced cells appeared to be in the ependymal wall, some of them appeared to be localized in the adjacent brain parenchyma (Fig. 6A arrow heads). These cells did not express HU antigen (Fig. 6B). Double labeling for PLAP (Fig. 6E) and GFAP (Fig. 6F) showed that the PLAP transgene was predominantly co-localized with GFAP in cells and processes (Fig. 6E and 6F). These data suggest that the PLAP expressing cells most probably correspond to ependymocytes, tanycytes and perhaps neural precursor cells interposed among the ependymocytes or in the subependymal zone [26-28]. In either case it is clear that the vector must infiltrate and permeate CSF efficiently, because no evidence of helper cells was seen in the brain parenchyma so far from the injection site in any of the control animals (e.g. Fig. 3H).

Several neural precursor cell populations may be susceptible to transduction by the dicistronic MLV vectors in the ventricular zone. Ependymal cells, which have been reported to be neural precursors [29], are still proliferating quite quickly in early postnatal brain [30]. Radial glia, which may be precursors of both neurons and glia [31-35], contact the ventricular surface and proliferate in the ventricular region until postnatal day 7 [36]. Lastly, the slowly proliferating GFAP-labeled subependymal neural stem cells, which survive and continue to proliferate and generate neurons in the adult brain, have been proposed to require contact with the ventricular surface to become neurogenic [27]. Having identified this sub-population of potential precursor cells we sought cells with mature phenotypes that may represent their progeny. In the most superficial layers of the cortex, which will be formed from the latest neurogenerative mitoses of cortical precursor cells, we were surprised to find rare transduced neurons, co-expressing PLAP and HU (Fig. 6H–I). Other non-neuronal cells labeled with the transgenes were also observed in forebrain (Fig. 6H,I,J and 6K).

These results showed that rather large numbers of transduced non-neuronal cells were found in and adjacent to the ependymal walls throughout the ventricular system including in the lateral ventricles (Fig. 6) and that the viral IRESes were active in these cells *in vivo*.

## Discussion

MLV-based double-IRES vectors pREV-HW3 and pEMCV-CBT4 were found to direct co-expression of two gene products in a variety of cell types [15,16,22,23]. Overall transgene expression driven from the MLV-based double-IRES vectors is the consequence of two distinct processes, transcription and translation initiation, both of which are tightly regulated by the host cell. Indeed, important limitations of MLV-vectors are cell-type-specific promoter silencing [13,37,38], and modulation of IRES activity. IRES activity can be regulated by diverse physiological processes such as cell cycle [39-42], cellular stress [43-46], cell transformation [47], cell death [48-51] and cell differentiation [52-56]. Previous studies suggested that the activity of the MLV IRES present in both vectors, could be modulated by oligodendrocyte differentiation [16]. The possibility of *in vivo *IRES regulation due to cell differentiation prompted us to extend our previous *ex vivo *studies [15,16], and evaluate the feasibility of using double-IRES MLV vectors in the CNS.

Results show that upon injection of producer cells in the cerebellum of newborn mice, the generated MLV-vectors transduce host cells throughout the postnatal brain ventricular system. Transduced neural precursors and their progeny could be revealed by histochemistry for PLAP or immunohistochemistry and large patches of transduced neurons could be identified expressing both transgenes 15 dpi. In double labeling studies, most cells that were PLAP positive also stained for Neo. Considering that MLV-vector producer cells appeared to survive less than 10 days in the developing brain these observations demonstrated transduction of proliferating precursor cells and the maintenance of IRES activity in neurons with each of the combinations of IRESes tested, namely MLV and REV-A (pHW3) and MLV and EMCV (pCBT4). Therefore, and consistent with previous observations [15,16], down-regulation of transgene expression was not observed in neurons generated from precursors transduced *in vivo*.

Transduced cells could be identified in the ependymal walls throughout the ventricular system. The 3^rd ^and lateral ventricles lie upstream of the injection site with respect to the flow of cerebrospinal fluid. Thus, the MLV vector is capable of diffusing via the cerebrospinal fluid and targets proliferating cells in this region of the brain. Diverse cell populations identified as sources of neuronal and glial precursors are potential targets for MLV-recombinant vector in early postnatal brain [27-29,34,35]. For example, the proliferation of ependymal cells, which form the interface between the CSF and the brain parenchyma, progressively slows down during postnatal development to a very low basal level at postnatal day 12 which then remains stable in the absence of injury [30,57]. Mitotic radial glia are also in contact with the ventricular surface in the lateral ventricles during early postnatal development [36]. Subventricular astrocytes also contact the ventricular surface in adult brain and proliferate slowly [27].

In the ventricular regions the majority of transduced cells express GFAP, which is is weakly expressed by ependymal cells and tanycytes and more strongly expressed by astrocytes and the GFAP labeled "type B" cells that constitute multipotent neural precursors [28,58]. Radial glia are also GFAP positive [59]. 15 dpi, transduced cells were observed in the brain parenchyma close to the ependymal wall. In the more superficial layers of the cortex, where the most immature post mitotic neurons reside, a few transduced neurons, identified by their expression of the HU antigen, as well as non-neuronal cells could be identified.

The current approaches for gene therapy of monogenetic diseases in mature organisms are confronted by several problems including: (1) adult tissues may be poorly infected by conventional vector systems dependent upon cell proliferation for optimal infection; (2) immune responses, whether pre-existing or developing after vector delivery, may rapidly eliminate transgenic protein expression and prevent future effective intervention. Early gene transfer, in the neonatal or even fetal period, may overcome some or all of these obstacles [60]. Therefore, the experimental approach described herein, might be useful in the development of new approaches to gene therapy in young organisms.

## Conclusion

In summary, we describe an eukaryotic dicistronic vector system that is capable of transducing mouse neural precursors *in vivo *andmaintaining the expression of genes after cell differentiation. Human placental alkaline phosphatase (PLAP) and neomycin phosphotransferase (Neo) used in this study as reporter genes can be replaced by other genes of interest to make these dicistronic vectors a novel tool to trace lineages and assess the function of specific genes in rodent CNS *in vivo*. Vectors might also be ideally suited to targeting suicide genes to proliferating cells, such as tumor cells, that spread and infiltrate via the CSF [61,62].

## Materials and methods

### Vectors, helper cells, titration

Plasmid vectors pEMCV-CBT4, pREV-HW1 and pREV-HW3, shown schematically in Fig 1, have been previously described [22,23]. NIH-3T3 cells, and the NIH-3T3 based retroviral packaging cell line GP+E-86 [63], were cultured in Dulbecco's modified Eagle's medium (DMEM, Gibco BRL) with 10% newborn calf serum at 37°C in presence of 5% CO_2_. MLV vectors were produced by transfection of GP+E-86 cells with pREV-HW3 or pEMCV-CBT4 constructs as previously described [22]. Vectors, produced by GP+E-86, were titrated on NIH-3T3 [15,16,22]. The negative control pREV-HW1 was produced using the same procedure as above.

### Grafts

Postnatal day 1–2 mice (OF1 strain) were injected with 1–5 × 10^4 ^producer cells in a 2 μl volume in the region of the developing cerebellum as follows. Producer cells harboring the recombinant vector, or control cells (non-transfected helper cells, transduced 3T3 cells or cells transfected with the pRev-HW 1 vector described by López-Lastra et al., (1997) which cannot be packaged, see Fig. 1), were resuspended by trypsinization, washed once in medium and twice in PBS, counted and resuspended in PBS. Cell suspension was pumped from a Hamilton syringe to fill a fine plastic catheter connected to a second Hamilton syringe needle. The second needle was manually pierced to a depth of 1.5 – 2 mm through the cranial cartilage into the region of the developing cerebellum, behind the cerebral hemispheres which were visible through the skull. Then, 2 μl of cell suspension was slowly pumped into the brain using the Hamilton syringe over a 10 second period. The needle was held in place for another 10 seconds and then carefully removed. The young were then replaced with their mothers and maintained with free access to food and water until the time of sacrifice. Animals were killed by anoxia in CO2, decapitated and their brains were carefully removed and fixed by immersion in 4% paraformaldehyde in PBS for 12 – 15 h. Brains were then cryoprotected by immersion in 30% sucrose and frozen by immersion in isopentane over dry ice. Brains were then cut into serial sections of 16 μμm thickness using a Leitz cryomicrotome and recovered on gelatin-coated glass microscope slides. All experiments involving animals were performed in accordance with the French regulations and were approved by the animal experimentation committee of the Ecole Normale Supérieure, Lyon.

### Histochemical staining

For placental alkaline phosphatase (PLAP) histochemical staining, cells were fixed in phosphate-buffered saline (PBS) containing 4% paraformaldehyde. After two washes in PBS, they were incubated at 65°C for 30 min in PBS. Tissue sections were incubated for 1 hour at 65°C. Cells or tissue sections were washed twice with AP buffer (100 mM Tris-HCl pH 9.5, 100 mM NaCl, and 5 mM MgCl_2_) and incubated for 5 hr in staining solution (0.1 mg/ml 5-bromo-4-chloro-3-indolyl phosphate (BCIP), 1 mg/ml nitroblue tetrazolium salt (NBT), and 1 mM levamisole) at 22°C. Brain regions of histochemically and immunostained cells were identified by extrapolation from a rat brain histological atlas [64].

### Immunohistochemistry

Tissue sections were rinsed in PBS then incubated for 30 min in 20 mM ammonium acetate. Sections were washed twice in PBS then incubated for 30 min in a blocking solution of PBS containing 5% BSA, 1% normal goat serum and 0.2% Tween 20 prior to staining with antibodies. This same solution served to dilute all the antibodies. Double-labelling was performed by simultaneous staining with antibodies produced in different species which were then revealed using fluorochrome-conjugated goat antibodies with appropriate species specificity. Thus, PLAP was revealed using a murine monoclonal antibody (diluted 1/200) purchased from DAKO (Glostrup, Denmark). Neo was revealed using an affinity purified rabbit polyclonal antibody (diluted 1/100) generated by immunizing rabbits with peptides VENGRFSGFIDCGRL and MIEQDGLHAGSPAAC conjugated by their carboxy terminus to keyhole limpet haemocyanin. GFAP which labels astrocytes [65], a population of neural stem cells [27,58] developing ependymocytes [58] and radial glia [59] was detected by a polyclonal rabbit anti-cow GFAP antiserum (diluted 1/200), purchased from DAKO. Neurons were detected using an anti-HU antiserum generously donated by Dr. J. Honnorat and Dr. M-F. Belin (diluted 1/1000). The HU antigen comprises a group of nucleic acid binding proteins located in the nucleus and cytoplasm of post-mitotic neurons [24]. All antibodies have been tested in cell culture and on various control tissues and give appropriate patterns of specific labeling. The neural cell type-specific markers did not label helper/producer cells *in vitro*. Primary incubations were for 2 h at room temperature or at 4°C overnight. After washing sections 5 × 10 min in PBS, bound antibodies were revealed with FITC-conjugated goat anti-human immunoglobulin antibodies or Cy3-conjugated goat anti-rabbit IgG antibodies and either Cy3- or FITC-conjugated goat anti-mouse IgG antibodies. Anti-immunoglobulin antibodies were all at a final dilution of 1/400 in blocking buffer containing bis-benzimide (1 μg/ml) to stain DNA. Controls included no primary antibodies and non-transduced brain. Slides were washed 3 times in PBS, mounted with moviol and analyzed with a Zeiss Axioplan fluorescence microscope.

## Authors' contributions

ED participated in the design of the study, conducted animal injection, maintained and handled animals, tissue sections, histochemical staining, immunohistochemistry, and drafted the manuscript. MLL participated in the design of the study, developed the retroviral vectors, generated the vector producing cell lines, aid in recombinant virus titration, and helped to draft the manuscript. JLD participated in the design of the study, was responsible for supervising and coordinating the study, and helped to draft the manuscript. All authors read and approved the final manuscript.
